# DNA Barcoding of Japanese Click Beetles (Coleoptera, Elateridae)

**DOI:** 10.1371/journal.pone.0116612

**Published:** 2015-01-30

**Authors:** Yuichi Oba, Hitoo Ôhira, Yukio Murase, Akihiko Moriyama, Yoshinori Kumazawa

**Affiliations:** 1 Graduate School of Bioagricultural Sciences, Nagoya University, Nagoya, 464-8601, Japan; 2 Kitsuneyama, Maigi-cho, Okazaki, 444-3511, Japan; 3 Research Center for Biological Diversity, Graduate School of Natural Sciences, Nagoya City University, Nagoya, 467-8501, Japan; Onderstepoort Veterinary Institute, SOUTH AFRICA

## Abstract

Click beetles (Coleoptera: Elateridae) represent one of the largest groups of beetle insects. Some click beetles in larval form, known as wireworms, are destructive agricultural pests. Morphological identification of click beetles is generally difficult and requires taxonomic expertise. This study reports on the DNA barcoding of Japanese click beetles to enable their rapid and accurate identification. We collected and assembled 762 cytochrome oxidase subunit I barcode sequences from 275 species, which cover approximately 75% of the common species found on the Japanese main island, Honshu. This barcode library also contains 20 out of the 21 potential pest species recorded in Japan. Our analysis shows that most morphologically identified species form distinct phylogenetic clusters separated from each other by large molecular distances. This supports the general usefulness of the DNA barcoding approach for quick and reliable identification of Japanese elaterid species for environmental impact assessment, agricultural pest control, and biodiversity analysis. On the other hand, the taxonomic boundary in dozens of species did not agree with the boundary of barcode index numbers (a criterion for sequence-based species delimitation). These findings urge taxonomic reinvestigation of these mismatched taxa.

## Introduction

Insects in the family Elateridae, commonly called click beetles, is one of the most highly diversified groups of the order Coleoptera. Approximately 10,000 [1,2] or even more [3] elaterid species are described worldwide, although an exact number in nature is uncertain owing to the frequent discovery of new species and frequent changes in taxonomy. Since most click beetles are dull in coloration and patterning and lack peculiar characteristics in, e.g., horn or large mandible, morphological identification is generally difficult and requires taxonomic expertise. While adults are typically nectar feeders, their larvae, called wireworms, usually live in the soil or under bark. Some wireworms are serious agricultural pests on, e.g., potato, wheat, and corn, while others are active predators of other insect larvae [1,2,4]. Identifying their larvae using morphological characteristics is even more difficult. The larval period for elaterids is generally long, often 2–3 years and sometimes 10 years or longer [2,5,6]. Thus, it is not practical to identify wireworm pests collected from crop fields following their development into adults. These features call the significance of DNA barcoding of click beetles to enable their easy, rapid and accurate identification.

**Figure 1:**
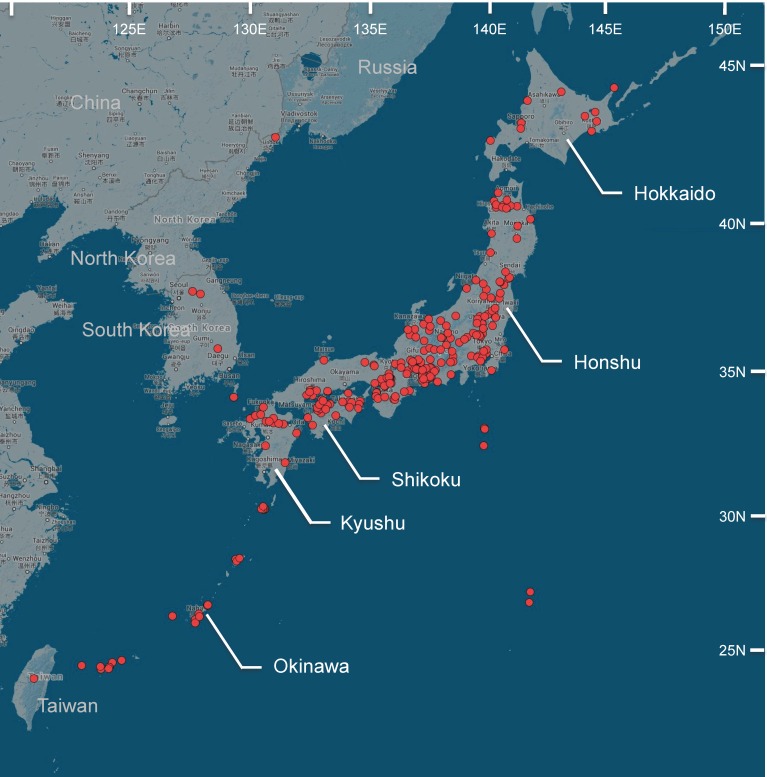
Map of Japan with sampling localities (red circles), created using Google Maps. Taxonomic lists of the Japanese elaterids have been published by some authors [11–13], and the number of species listed has steadily increased (Table 1). At present, approximately 130 elaterid genera and 770 species have been recorded in Japan (reviewed in Ôhira, 2013 [3]), although the most updated species list was not published with this review. This number of elaterid species in Japan is comparable with that of the whole Nearctic region (965 species) [2] and much larger than that of the British Islands (73 species) [14], which are as large as the Japanese Islands. These numbers highlight the high elaterid biodiversity in Japan. Adult elaterids are relatively common in Japan. They are often collected in various traps during environmental impact statement research and thus appear as significant members in the prefectural lists of wild insects. For example, Elateridae constitutes 5.1% and 4.9% of the beetle species collected in Tochigi [15] and Okayama [16] prefectures, respectively.

The Japanese archipelago consists of four main islands (Hokkaido, Honshu, Kyushu, and Shikoku) and ∼3000 smaller islands (Fig. 1) that stretch from the humid subtropics in the south to the boreal zone in the north, and includes a wide variety of climates and ecosystems. Forests occupy two-thirds of the Japanese land area, ranging from subalpine to coastal zones [7]. After the Miocene period, Japan has repeatedly undergone connection/disconnection to/from Eurasia. As a result, Japan is one of the biologically richest terrestrial ecoregions on earth [8,9], and is one of the world’s 34 “Biodiversity Hotspots” [10].

**Table 1:** Taxonomic coverage of DNA barcoding for Japan and the Japanese regions.

**Country/Region/Prefecture**	**Barcoded (genus/species)**	**Listed in reference (genus/species)^a^**	**Taxon coverage (genus/species)**	**Reference**
Japan (378,000 km^2^)	89/184	106/277^c^	83%/65%	[11]
	92/236	109/515	84%/46%	[12]
	86/257	117/639	74%/40%	[13]
Hokkaido main island (83,500 km^2^)	59/106**^b^**	65/161	91%/66%	[44]
Honshu main island (278,000 km^2^)	72/134	80/181^c^	90%/74%	[11]
Miyagi Pref. (7,300 km^2^)	50/76**^b^**	51/89	98%/85%	[45]
Tochigi Pref. (6,400 km^2^)	60/137**^b^**	65/181	92%/76%	[15]
Kanagawa Pref. (2,400 km^2^)	60/118**^b^**	64/154	94%/77%	[46]
Fukui Pref. (4,200 km^2^)	60/119**^b^**	63/156	95%/76%	[47]
Aichi Pref. (5,200 km^2^)	61/121**^b^**	63/145	97%/83%	[48]
Kyoto Pref. (4,600 km^2^)	59/107**^b^**	61/135	97%/79%	[49]
Okayama Pref. (7,100 km^2^)	65/126**^b^**	69/171	94%/74%	[16]
Okinawa Pref.+ Satsunan Islands (4,600 km^2^)	53/78**^b^**	63/170	84%/46%	[50]
Okinawa Pref. (2,300 km^2^)	50/69**^b^**	60/166	83%/42%	[51]

^a^Numbers of genus and species in reference lists are modified based on current taxonomy.

^b^Numbers include specimens collected from other prefectures or regions.

^c^Ôhira and Suzuki [11] primarily list common species of Japanese elaterids but include some rare species as well.

Serious agricultural damages by wireworms have also been reported in Japan (S1 Table). For example, *Melanotus fortnumi fortnumi* is a common pest on wheat, corn and sweet potato [17–20]. *Agriotes ogurae fuscicollis* and *Melanotus okinawensis* are also known as pests on corn, oats and potato [21] and on sugarcane [22], respectively. Some species are endangered and thus appear on ‘Red Lists of threatened species’ of some prefectures. For example, *Aganohypoganus mirabilis* and *Sadoganus babai* are listed as Vulnerable (VU) in Aichi Prefecture [23].

In this study, we report on 762 DNA barcodes collected and assembled from 90 genera and 275 species, including most of the potential agricultural pest species (20 out of 21 species, S1 Table) and threatened species, which collectively cover approximately 75% of the common species that occur on Honshu main island (Table 1). We evaluate the usefulness of this barcode database for efficient and reliable species identification. We also provide perspectives into how the DNA barcodes will inform taxonomic reconsideration of Japanese elaterids. As several Elateridae genera in Japan occur in other countries, including North America and Europe, our database of the Japanese click beetles should also be useful for taxonomists and ecologists of other countries.

## Materials and Methods

### Specimens

Adult elaterids were collected during 2003–2012, mainly from Japan (Hokkaido, Honshu, Shikoku, Kyushu, Okinawa and their adjacent islands; Fig. 1) using beating sheets, light traps, flight interception traps, bait traps, and other sampling methods. Some specimens were collected from neighboring countries to clarify the taxonomic status of the Japanese species by comparison with closely related foreign species. All sampling activities were performed at locations where specific permission is not required, and the specimens did not include endangered or protected species. All specimens were identified morphologically by HÔ. Collected specimens were directly soaked into 99.5% ethanol and stored at 4°C. Genitalia dissection and preparation were performed when necessary to validate identification of the specimens. Ethanol-preserved voucher specimens are deposited in the Specimen Depository of the Graduate School of Natural Sciences, Nagoya City University (SDNCU) with voucher numbers shown in S1 Table.

### DNA extraction, amplification and sequencing

Whenever possible, we collected and analyzed multiple individuals from each species (or subspecies). However, for each species, only one specimen per locality was included in this study. Total DNA was extracted from legs of a single specimen as previously reported [24]. Legs dissected from ethanol-preserved specimens were homogenized in TNES-Urea buffer (8 M urea, 30 mM Tris-HCl, 125 mM NaCl, 10 mM EDTA, 1% SDS, pH7.5) and treated with proteinase K at 37°C. The mixture was extracted with phenol-chloroform (1:1) and chloroform-isoamyl alcohol (24:1). Total DNA was precipitated with ethanol and dissolved in Tris-EDTA buffer.

The ‘barcode’ region of cytochrome oxidase subunit I gene (*COI*) was amplified using SpeedSTAR HS DNA polymerase (Takara, Shiga, Japan). In addition to the standard primers for DNA barcoding (LCO1490 and HCO2198) [25], we designed four primers that served to amplify sequences encompassing the barcode region between LCO1490 and HCO2198 (Table 2). The barcode region of most *Homotechnes* specimens was not amplified with standard primers LCO1490 and HCO2198, but was with primers eTyr-1H and eCO1-2H (Table 2).

**Table 2:** Primers used to amplify and sequence the *COI* barcode region.

**Name**	**Primer sequence**	**Direction**	**Reference**
LCO1490	5′–GGT CAA CAA ATC ATA AAG ATA TTG G–3′	forward	[25]
HCO2198	5′–TAA ACT TCA GGG TGA CCA AAA AAT CA–3′	reverse	[25]
eCO1-1L	5′–AAA TGA TTA TTT TCA ACA AAC CAT AAA–3	forward	This study
eTyr-1L	5′–ATC GCC TAA ACT CAG CCA TCT TAC T–3′	forward	This study
eCO1-1H	5′–ACA ATG TGA GAG ATT ATT CCA AAT CC–3′	reverse	This study
eCO1-2H	5′–CCT AGG AGT CCA ATT GCT ATT ATA GC–3′	reverse	This study

The PCR reaction was performed using a Dice TP 650 thermal cycler (Takara) with the following cycle conditions: 35 cycles of 98°C for 5 sec, 50°C for 15 sec, and 72°C for 20 sec. Nested PCR was not performed to avoid false amplification. PCR products were cleaned up with an exonuclease I and shrimp alkaline phosphatase (ExoSAP-IT; Affymetrix, Cleveland, OH) and directly sequenced using a BigDye Terminator v3.1 Cycle Sequencing Kit (Applied Biosystems, Foster City, CA) and an Applied Biosystems 373A or 3500 Genetic Analyzer (Life Technologies). Sequences from both directions were assembled with Sequencher v4.8 (Gene Codes, An Arbor, MI) to generate the barcode sequence for each specimen. All specimens were labeled with accompanying information on the sampling date and locality, and photographed with a scale from multiple directions. These information are publicly available at the DDBJ/EMBL/GenBank nucleotide sequence databases (accession numbers, KM612281 – KM613042), Barcode of Life Data Systems (BOLD) [26], and Japanese Barcode of Life Database (JBOL-DB; http://db.jboli.org/) under the ‘Japanese Elateridae Barcoding Project (JEBP).’

### Data analysis

Sequence divergence was calculated using *p*-distance, and multiple alignment was generated by the BOLD sequence aligner. A phylogenetic tree using barcode region sequences (658 bp) was constructed under the Kimura 2-parameter model using the neighbor-joining algorithm in the BOLD system. Other sequence analyses were performed using the ‘Distance Summary’, ‘Barcode Gap Analysis’, and ‘BIN Discordance Report’ options in the BOLD Workbench with the ‘complete deletion’ mode for handling gap-containing sites.

## Results

### Genetic variation

**Figure 2:**
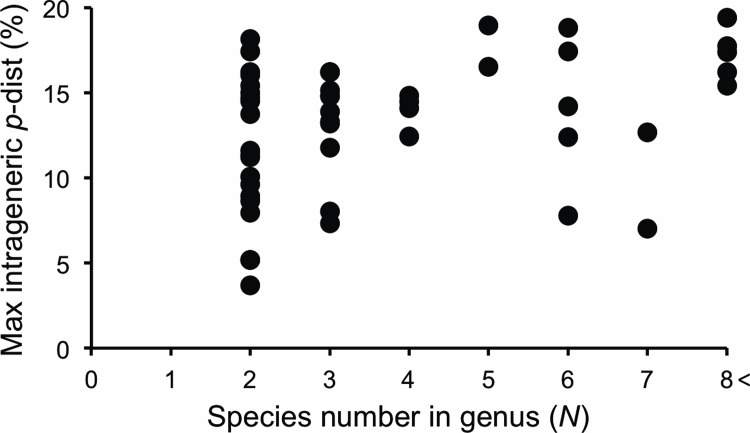
Maximum *p*-distance for genus vs. the number of species analyzed. No strong correlation was found between the two variables (Spearman’s rank correlation coefficient *r_s_* = 0.356).

A total of 762 *COI* barcode sequences (≥ 658 bp) were generated in this study from 275 species and 90 genera. Analysis was performed on 1–68 specimens per species (2.8 on average). Seven hundred fifty sequences (98.4%) were derived from Japanese specimens, and the others were from neighboring countries such as Taiwan, China, Russia, Korea and Thailand. All DNA barcodes encompassed the full 658 bp ‘barcode’ region with high trace file qualities. The BOLD system indicated that 81% of barcodes have ‘high’ quality and that 13% have ‘medium’ quality. The mean and maximum of intraspecific *p*-distance were 3.7% and 13.5%, respectively (Table 3). This mean value is relatively high compared to the corresponding value for elaterid species in North Europe according to Pentinsaari et al. [27](0.43%), while the maximum intraspecific *p*-distance of *COI* was similar between Japanese (13.5%; Table 3) and North European elaterids (12.36%; [27]). The average *p*-distance between congeneric species of the Japanese elaterids was 11.6% (Table 3). Hebert et al. [28] found the mean *p*-distance of the *COI* barcode region among 13,320 congeneric species in 11 phyla to be 11.3%. Staudacher et al. [29] analyzed Elateridae DNA barcodes of 53 individuals of 17 *Agriotes* species from Central Europe and found a mean interspecific *p*-distance of 11.4%. Thus, Japanese elaterids show a similar average *p*-distance between congeneric species compared with those of previous studies. Maximum distances between congeneric species were not significantly (*p* < 0.01) dependent on the number of species in a genus based on Spearman’s rank correlation coefficient, indicating no strong sampling bias in our analysis (Fig. 2). Table 3 indicates that most pairs of Japanese elaterid species are separated by a sufficient number of diagnostic substitutions, as shown for many other taxa [28].

**Table 3:** Distribution of sequence divergence (K2P distance/uncorrected *p*-distance).

	**Individuals**	**Taxa**	**Min Dist %**	**Mean Dist %**	**Max Dist %**
Within species	622	138	0.0/ 0.0	3.9/ 3.7	15.0/13.5
Within genus	680	49	0.0/ 0.0	12.8/ 11.6	22.7/19.4
Within family	756	1	5.1/ 4.9	20.6/ 17.9	32.8/26.0

Phylogenetic analysis (S1 Fig.) showed that a majority of genera containing multiple species form monophyletic groups (35 out of 49 genera as summarized in S2 Table). While the genera *Agrypnus* and *Hemicrepidius* are polyphyletic, subgenera of each genus are monophyletic (S2 Table). This congruence between taxonomic and molecular status of these genera (or subgenera) suggests that elaterid specimens from Japan are generally identifiable at the genus level even if the query species is not included in the reference database. However, several genera are paraphyletic and nest the other genera (S1 Fig. and S2 Table): e.g., *Reitterelater* nested in *Ampedus, Corymbitode*s nested in *Acteniceromorphus, Parasilesis* nested in *Glyphonyx*, and *Babadrasterius* nested in *Heteroderes*. Indeed, close relationships of these genus pairs are in agreement with a different molecular analysis using nuclear 28S ribosomal DNA [30,31].

### Barcode gap and barcode index number

**Figure 3:**
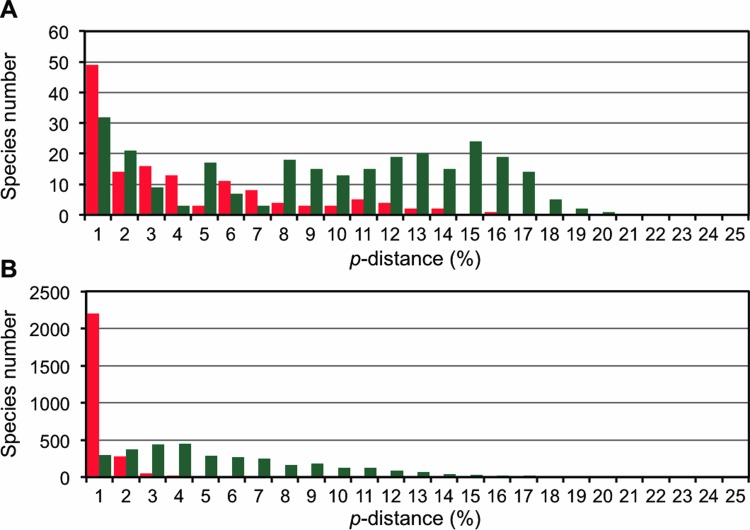
Distributions of the maximum intraspecific *p*-distances (red) and the nearest neighbor distance for each species (green). Sequences below 500 bp were eliminated. (A) Japanese click beetles (project name, JEBP). (B) Combination of plots from eight public DNA barcoding datasets (see S4 Fig. for plots of each of the eight datasets) that were used to describe the BIN system by Ratnasingham and Hebert [32]. The public data were acquired from the BOLD system on 7 April 2014.

The Barcode Gap Analysis in the BOLD system provides the distribution of distances within each species and the distance to the nearest neighbor of each species (Fig. 3), so that species are tested for the presence of the ‘Barcode Gap’ (2% *COI* divergence is considered the empirical criterion for the species boundary; [32] and refs. therein). This analysis showed that 211 species (77% of all species analyzed) are separated from their sister species by the barcode gap. However, 53 species have a less than 2% distance to their sister species and 37 species have lower distances to their nearest neighbor than their maximum intraspecific distances and are therefore tagged as ‘warning’ for the Barcode Gap application. Note that 64 species in total were warned by one or both of these criteria. These ‘warning’ species include taxonomically controversial taxa as noted in Table 4. In the genus *Ampedus*, for example, large numbers of species (∼130 species) have been recorded from various regions of Japan but their species delimitations are sometimes ambiguous [3]. Congeneric species in *Dalopius* and *Paracardiophorus* from Japan are also very similar to each other in external morphology and are sometimes difficult to identify, making the taxonomy of these genera traditionally unstable [33,34].

**Table 4:** ‘Warning’ species tagged by the BOLD Barcode Gap Analysis with references for their taxonomic problems.

**Genus**	**Species**	**Max intraSP dist.**	**NN**	**Dist to NN**	**Reference**
*Actenicerus*	*giganteus*	3.17	*toyoshimai*	1.71	[35]
	*kiashianus*	4.29	*yamashitai*	0.46	[35]
	*kidonoi*	3.00	*orientalis*	0.15	[35]
	*nempta*	N/A	*orientalis*	1.40	[35]
	*orientalis*	9.11	*kidonoi*	0.15	[35]
	*toyoshimai*	N/A	*orientalis*	0.46	[35]
	*yamashitai*	N/A	*kiashianus*	0.46	[35]
*Agrypnus*	*binodulus*	11.76	*scutellaris*	9.57	-
	*miyamotoi*	11.76	*tsukamotoi*	0.31	[40]
	*scrofa*	3.48	*tsukamotoi*	0.15	[40]
	*tsukamotoi*	10.07	*tsushimensis*	0.00	[40]
	*tsushimensis*	5.95	*tsukamotoi*	0.00	[40]
*Ampedus*	*aureopilosus*	N/A	*convexicollis*	0.46	[3]
	*carbunculus*	0.00	*ohtsukai*	0.77	[3]
	*chokai*	N/A	*tokachi*	1.87	[3]
	*convexicollis*	N/A	*aureopilosus*	0.46	[3]
	*hypogastricus*	2.99	*vestitus*	0.77	[3]
	*ohdai*	N/A	*tokugoensis*	1.56	[3]
	*ohtsukai*	N/A	*carbunculus*	0.77	[3]
	*oiwakensis*	N/A	*tenuistriatus*	1.40	[3]
	*tenuistriatus*	2.99	*tokachi*	0.31	[3]
	*tokachi*	N/A	*tenuistriatus*	0.31	[3]
	*tokugoensis*	N/A	*ohdai*	1.56	[3]
	*vestitus*	2.34	*hypogastricus*	0.77	[3]
*Ascoliocerus*	*fluviatilis*	N/A	*saxatilis*	0.31	-
	*saxatilis*	5.46	*fluviatilis*	0.31	-
*Dalopius*	*ainu*	N/A	*mutsuensis*	1.40	[3,33]
	*exilis*	N/A	*mutsuensis*	1.09	[3,33]
	*mutsuensis*	N/A	*exilis*	1.09	[3,33]
	*patagiatus*	4.48	*tamui*	1.24	[3,33]
	*tamui*	N/A	*patagiatus*	1.24	[3,33]
*Ectinus*	*longicollis*	5.62	*hidaensis*	2.67	-
*Fleutiauxellus*	*curatus*	9.73	*niponicus*	8.51	-
*Hemicrepidius*	*secessus*	6.01	*sinuatus*	0.46	[52]
	*sinuatus*	2.35	*secessus*	0.46	[52]
*Homotechnes*	*brunneofuscus*	6.85	*motschulskyi*	2.67	-
	*motschulskyi*	7.86	*brunneofuscus*	2.67	-
*Limoniscus*	*niponensis*	N/A	*yamato*	0.15	[53]
	*yamato*	0.62	*niponensis*	0.15	[53]
*Melanotus*	*correctus*	10.30	*ocellatopunctatus*	0.00	[54]
	*erythropygus*	12.74	*fortnumi*	1.87	[55]
	*fortnumi*	5.78	*niponicus*	1.08	[55]
	*legatus*	12.09	*yaeyamacola*	5.80	-
	*niponicus*	N/A	*fortnumi*	1.08	[55]
	*ocellatopunctatus*	N/A	*correctus*	0.00	[54]
*Nipponoelater*	*babai*	7.32	*amami*	2.34	-
*Oedostethus*	*kohnoi*	N/A	*ozakii*	1.71	-
	*ozakii*	N/A	*kohnoi*	1.71	-
*Paracardiophorus*	*lewisi*	9.20	*sequens*	2.83	[24]
	*nakanei*	3.31	*pullatus*	0.31	[24]
	*opacus*	N/A	*pullatus*	1.56	[24]
	*pullatus*	11.56	*nakanei*	0.31	[24]
	*sequens*	5.48	*pullatus*	0.62	[24]
*Parasilesis*	*musculus*	8.05	*shirozui*	0.62	[56]
	*shirozui*	N/A	*musculus*	0.62	[56]
*Pectocera*	*fortunei*	7.33	*amamiinsulana*	4.46	[57]
*Platynychus*	*nothus*	11.99	*formosanus*	9.05	[3]
*Priopus*	*ferrugineipennis*	6.87	*yonaguni*	4.12	-
*Sericus*	*brunneus*	N/A	*fugisanus*	1.55	-
	*fugisanus*	N/A	*brunneus*	1.55	-
*Vuilletus*	*crebrepunctatus*	10.03	*viridis*	1.40	[3,58]
	*viridis*	10.41	*crebrepunctatus*	1.40	[3,58]
*Zorochros*	*albipilis*	N/A	*osawai*	0.15	[59]
	*osawai*	N/A	*albipilis*	0.15	[59]

N/A represents the singleton species.

The BOLD system implements an advanced protocol, named Barcode Index Number (BIN) system for the DNA-based delineation of species [32]. Apart from the Linnaean species name, the BIN system generates presumptive species codes (BINs) using a clustering process with the 2% ‘barcode gap’ criterion followed by cluster refinement using the Markov clustering and Silhouette criterion [32]. This analysis revealed that 762 elaterid *COI* sequences were grouped into 389 BINs (S1 Table), of which 136 BINs (429 sequences) were taxonomically concordant (i.e., BINs having multiple individuals of a single named species, permitting multiple BINs to correspond to a species), 36 BINs (116 sequences) were discordant, and 217 BINs (217 sequences) were singletons (i.e., BINs having a single individual, permitting multiple BINs to correspond to a species).

**Figure 4:**
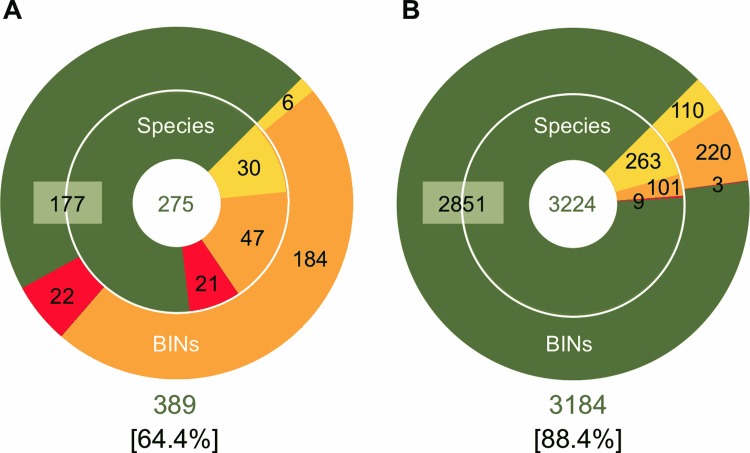
Comparisons between species and BIN boundaries. Numbers represent species (inner ring) and BINs (outer ring) categorized into MATCHES (green), MERGES (yellow), SPLITS (orange) or MIXTURES (red). (A) Elaterid dataset of this study. (B) Combination of eight public barcoding datasets for bees, birds, fishes, butterflies and moths (after Ratnasingham and Hebert, 2013 [32]). In a bracket below each chart, the percentage of species that are MATCHES is shown.

Association between BINs and Linnaean species can also be categorized into four patterns: MATCHES, one species corresponds to one BIN, and *vice versa*; SPLITS, one species corresponds to multiple BINs; MERGES, multiple species correspond to one BIN; and MIXTURES, a mixture of SPLITS and MERGES [32]. With our elaterid barcoding data, 177 species matched their corresponding BINs, 30 species were merged into six BINs, 47 species were split into 184 BINs, and finally 21 species showed a mixture of SPLITS and MERGES (Fig. 4A). These results indicate that species boundaries in approximately 36% of elaterid species do not seem to match well to those inferred from the BIN system.

The following sections describe several examples of major discrepancies between the Linnaean and BIN systems.

### Melanotus legatus

*Melanotus legatus legatus* is one of the most common elaterid subspecies in Japan, distributed widely on Hokkaido, Honshu, Shikoku and Kyushu main islands and adjacent islands. The BOLD BIN system indicated two distinct BINs for *M. legatus legatus* (10.6–11.0% *p*-distance between them, and 0.0–1.7% *p*-distance within each BIN) without geographic separation of the corresponding individuals. The genetic distance between 13 individuals of one BIN (ACI7154) containing both male and female was zero, while another BIN (ACI7155) was more closely related to two congeneric BINs representing *M. legatus masakianus* and *M. yaeyamacola* than to ACI7154 (Table 4 and S1 Fig.).

### Agrypnus binodulus

*Agrypnus binodulus binodulus* is also one of the most common subspecies in Japan, distributed widely on Hokkaido, Honshu, Shikoku and Kyushu main islands and adjacent islands. This taxon was divided into three BINs (one of which is a singleton; S1 Table) (7.0–9.3% *p*-distances between BINs, and 0.3–3.2% *p*-distances within them) with the distributions of the corresponding individuals overlapping between BINs. Two Korean specimens of *A. binodulus coreanus*, which is distributed in Korea, Northeast China and Tsushima Islands of Japan, were given an independent BIN (ACI6975, S1 Table), and the genetic distance (*p*-distance) between the individuals of *A. binodulus binodulus* and *A. binodulus coreanus* was 7.6–10.7%. This value is comparable to those among three BINs of *A. binodulus binodulus*. Distance to the nearest neighbor species *A. scutellaris* (9.6%) was lower than the maximum intraspecific distance within *A. binodulus* (11.8%; Table 4). These data suggest that *A. binodulus binodulus* can be divided into multiple species.

### Pectocera fortunei

*Pectocera fortunei fortunei*, an additional common subspecies, is distributed on flatlands of Honshu, Shikoku and Kyushu main islands and adjacent smaller islands. The BOLD BIN system indicated three BINs (S1 Table) in *P. fortunei fortunei* (2.4–6.9% *p*-distances between BINs, and 0.0–1.4% *p*-distances within a single BIN) and the distribution of the individuals overlapped between them (S2 Fig.). *P. fortunei* nested six other species, *P. amamiinsulana, P. maruyamai, P. yonaha, P. formosana, P. yaeyamana*, and an undescribed *P*. sp. (distributed in Korea) in the phylogenetic tree (S1 Fig.). In addition, *P. fortunei* had a lower distance to *P. amamiinsulana* than their maximum intraspecific distances (Table 4).

### Actenicerus

In the genus *Actenicerus*, 37 species have been found worldwide including in the Nearctic Region, Europe, Russia, East and Southeast Asia. Of these 26 are recorded as endemic species in Japan [3,35]. Thus, *Actenicerus* is particularly diversified in Japan [35–37]. In this study, we analyzed 79 *Actenicerus* barcodes consisting of 18 species from Japan and two species from Taiwan and Russia. These 79 individuals were grouped into 36 BINs (18 concordant, 4 discordant and 14 singleton BINs; S1 Table and S3 Fig.). It is noteworthy that the individuals identified as *Actenicerus orientalis*, named the ‘*orientalis* group’ by Sagegami-Oba et al. [38], nested six species (*A. kidonoi, A. giganteus, A. naomii, A. alternatus, A. nempta*, and *A. toyoshimai*) in the phylogenetic tree (S1 Fig.). *A. orientalis* is distributed in a clumped pattern, mostly in disjunct montane regions and has some morphological variations [36,39]. There were large molecular distances between lineages of the ‘*orientalis* group’ (maximum 9.4%), while distances of *A. toyoshimai, A. nempta* and *A. kidonoi* to their sister lineages of *A. orientalis* were small (0.5%, 1.4% and 0.2%, respectively; Table 4). These results suggest that the taxonomic status of these species should be reconsidered.

### Homotechnes motschulskyi

*Homotechnes motschulskyi* is flightless owing to degenerated hindwings. This species is patchily distributed in the high mountains of Honshu and Shikoku main islands. Since unique morphological characteristics have been recognized for each of these populations, *H. motschulskyi* is currently divided into 60 subspecies [3]. In the present study, we analyzed 68 *H. motschulskyi* individuals, consisting of 38 subspecies (including six unknown, probably novel, subspecies). As a result, the 68 barcodes showed 0.0–7.5% *p*-distances (3.9% in mean), being grouped into 39 BINs (of which 23 were singletons; S1 Table). This large BIN number is incidentally very close to the subspecies number (*N* = 38), but they do not correspond to each other very well (only 14 out of 32 identified subspecies are in the MATCHES category). It is of note that *H. motschulskyi* has a lower distance to the winged *Homotechnes brunneofuscus* compared with their maximum intraspecific distance (Table 4).

### Colaulon

In the subgenus *Colaulon* of the genus *Agrypnus*, many of the species have degenerated hindwings and are flightless. These species are mainly distributed in small clumped populations in discontinuous coastal areas [35]. Probably because of this patchy distribution, morphological diversity has been recognized among these flightless taxa. Although eight species and 17 subspecies have been described in the subgenus *Colaulon* in Japan, their taxonomy is still confused [40]. In the present study, we analyzed five species and two subspecies (*N* = 18) of *Colaulon*. The BIN analysis showed that all analyzed species were in the MIXTURES category except for winged *A. hypnicola* (S1 Fig.), implying recent events of mtDNA introgression, speciation and/or parallel adaptation changes in morphology of *Colaulon* species in Japan.

### Endemism

As of March 2014, the BOLD system included 129 registered BINs of Elateridae for specimens collected mostly in European and North American localities. Only six out of the 129 BINs overlapped with the 389 BINs of the Japanese species (asterisks in S1 Table), suggesting high endemism of elaterid species in general. Okinawa is the southernmost prefecture of Japan, comprising hundreds of islands. In contrast to the temperate climate in most Japanese areas, Okinawa is recognized as a subtropical region. Our data contain 61 individuals of 46 species from Okinawa, and none of these share the same BINs with those from the other Japanese areas (S1 Fig.). This result suggests high endemism of elaterid species in Okinawa. Hokkaido is the northernmost prefecture of Japan. Its climate is recognized as subarctic. Our data contain 42 individuals of 37 species from Hokkaido. In contrast to the case of Okinawa, 25 out of the 42 individuals share the same BINs with those from the other Japanese areas (S1 Fig.). Note that the following four species are supposed to be endemic to Hokkaido but that their individuals share the same BINs with different species in Honshu: *Melanotus ocellatopunctatus, Dalopius ainu, Limoniscus niponensis* and *Ampedus tokachi* (see Table 4).

## Discussion

In this study, we assembled 762 DNA barcodes for Japanese click beetles (S1 Table), covering 74% of the common species (90% in genus) that occur on Honshu main island and 74–85% of recorded species (94–98% in genus) from several prefectures of Honshu (Table 1). Since regional Red Listing process and environmental impact assessment are usually conducted on the basis of the prefectural species lists, high coverage of barcoded species in the prefectural species list is important for practical use. All 762 individuals were carefully identified by a taxonomic expert on Elateridae (HÔ). Thus, our database satisfies the two most important factors for DNA barcode libraries, coverage and reliability [41].

Of 389 BINs detected, 91% were concordant or singletons. Most genera or subgenera form a monophyletic clade in the neighbor-joining tree (S2 Table), and the average *p*-distance between congeneric species (11.6%) was much higher than that within species (3.7%) (Table 3). These results lend support to the general usefulness of the JEBP database in identifying elaterid specimens. Even when the query species was not represented in the reference database, it could be identifiable at least to the genus level.

However, we also detected a number of discrepancies between morphology-based traditional taxonomy and DNA-based phylogenetic clusters (Fig. 4) [32]. The proportion of MATCHES species for Japanese Elateridae (64%; Fig. 4A) was considerably smaller than that for eight other animal groups ranging from birds and fishes to bees and moths (83–97% [32], 88% on average; Fig. 4B and S2 Fig.). *Ampedus* species are major elaterid taxa that fell into the category of MERGES (30 species), while *Homotechnes motschulskyi* and *Actenicerus* species are major taxa in the SPLITS category (47 species) (S1 Table). To evaluate the contribution of specific taxa in reducing the MATCHES proportion for the Japanese Elateridae, we recalculated the proportions of MATCHES/SPLITS/MERGES/MIXTURES without the dataset for *Ampedus, Actenicerus*, and *H. motschulskyi* species. The result showed that the MATCHES proportion only slightly increased (69%), suggesting that the discrepancy between morphology-based traditional taxonomy and DNA-based phylogenetic clusters in the Japanese Elateridae is not fully attributable to a particular taxon. Moreover, as outlined earlier, several other taxa (e.g., *Melanotus legatus, Agrypnus binodulus, Pectocera fortunei*, and *Colaulon*) show mismatches to their BINs in different ways. These problematic taxa can be found commonly in Japan, but their taxonomy is confused. Since there are still a number of controversial issues regarding the taxonomy of Japanese click beetles, the barcode data presented in this report are expected to provide an objective criterion for their taxonomic revision.

In principle, reasons for the discrepancies between current taxonomy and BIN assignments could be 1) immaturity of taxonomic investigation, 2) sequencing errors by, e.g., sample contamination, and 3) the inability of *COI* sequence variations to appropriately diagnose species due to, e.g., stochastic errors, aberrant *COI* gene evolution, gene introgression, and very recent speciation. As noted above, we suspect that taxonomic immaturity for Japanese elaterids, partly due to the morphological resemblance among species, is a major reason for the discrepancies in the present analysis. On the other hand, we are very confident in the accuracy of our sequencing results. During the experiments, sequence contaminations did occasionally occur and phylogenetic placement of the corresponding individuals was unreasonable. When there was even a small doubt in the accuracy of sequencing results, we conducted additional amplification and sequencing with different primers or sequencing of a second individual. Some of the apparently unreasonable phylogenetic placements were also ascribed to identification errors, which were corrected accordingly. Careful operation of these checking cycles significantly increased the quality of our barcoding database.

Regarding the third possible reason stated in the last paragraph, Fig. 3 seems to show an intriguing pattern of distance distributions. As shown in Fig. 3B and S4 Fig., the Barcode Gap (2% distance) usually exists between distributions of the maximum intraspecific *p*-distances for each species and the nearest neighbor *p*-distances for each species. However, the Japanese elaterids appear to show an elevated gap level (5–10% distance) in Fig. 3A. Again, we suspect that this is largely an artifact due to taxonomic immaturity. However, we cannot strictly discount the possibilities of increased rates in *COI* sequence evolution and/or decreased rates of speciation in Elateridae. To the best of our knowledge, there is no evidence of changes in these rates for elaterids. Furthermore, they are quite a diversified group and there is no circumstantial evidence for any decline in the speciation rate.

Click beetles are economically important insects. Adults are common in Japanese fauna and many individuals can be encountered in the field. The soil-dwelling larvae (wireworms) are well-known as agricultural pests on various crops and vegetables (S1 Table). Various methods have been studied for controlling and monitoring wireworms in agricultural fields, including mating disruption or trapping by species-specific sex pheromones [42,43]. Evaluation of pheromone effects requires correct identification of individual click beetles. However, identification of adult click beetles requires considerable skill and taxonomic expertise. Identification of wireworms is even more difficult because there is little knowledge of larval morphology [3,6]. Identification of elaterids by the traditional morphological approach will be increasingly difficult because there are now very few specialists for click beetle taxonomy in Japan and this situation will not change in the near future. Consequently, an alternative method for rapid and precise identification of click beetles is desired. In this context, the JEBP database described in this report will be a valuable tool for rapid and reliable identification of the Japanese click beetles, even at larval stages. We have already applied this approach and have identified *Melanotus annosus* as an adult pest of persimmon fruits, a conclusion confirmed by analysis of morphological characteristics including male genitalia (Oba et al., unpublished data).

The present work, conducted as part of the Japanese Barcode of Life Database (JBOL-DB) project, is the first comprehensive barcode analysis of Japanese elaterids. We hope that more efforts will be made in the future to catalog insect life in Japan, one of the world’s biodiversity ‘hotspots’.

## Supporting Information

S1 Fig Neighbor-joining tree constructed using 762 *COI* barcode sequences of Elateridae. Species (and subspecies) name|Sample ID|Prefecture (or country) are shown.
(PDF)

S2 Fig Map of collection localities with sample IDs for *Pectocera fortunei* analyzed in this study. (PDF)

S3 Fig Map of collection localities with sample IDs and BINs for the *Actenicerus* ‘*orientalis* group’ analyzed in this study. (PDF)

S4 Fig Distributions of maximum intraspecific *p*-distance (red) and the nearest neighbor distance for each species (green). Sequences below 500 bp were eliminated. Eight public DNA barcoding datasets were chosen following Ratnasingham and Hebert, 2013 [32]. DS-AVESNT1, dataset of Neotropical birds (497 species); DS-AVESNA1, dataset of North American birds (575 species); DS-BEEIRE1, dataset of Irish bees (58 species); DS-FISHAUS1, dataset of Australian fishes (214 species); DS-FISHCAN1, dataset of Canadian fishes (190 species); DS-GEOBAV1, dataset of German geometer moths (301 species); DS-LEPNA1, dataset of North American moths and butterflies (1347 species); and DS-PLUSNA1, dataset of North American noctuid moths in the Plusiinae subfamily (71 species). Public data were acquired from the BOLD system on 7 April 2014.
(PDF)

S1 Table Data of specimens used in this study. (XLSX)

S2 Table List of the genera analyzed in this study. (XLSX)
