# Correction: DNA Barcoding of Japanese Click Beetles (Coleoptera, Elateridae)

**DOI:** 10.1371/journal.pone.0124857

**Published:** 2015-04-09

**Authors:** The PLOS ONE Staff

During typesetting, the third paragraph of the Introduction was erroneously included in the caption for Fig 1. The publisher apologizes for this error. The unbolded text in the caption for Fig 1, with the exclusion of “doi:10.1371/journal.pone.0116612.g001,” should be the third paragraph of the Introduction, which should appear before Table 1 and after Fig 1. Please view the correct Fig 1, renamed Fig 1, below.

**Fig 1:**
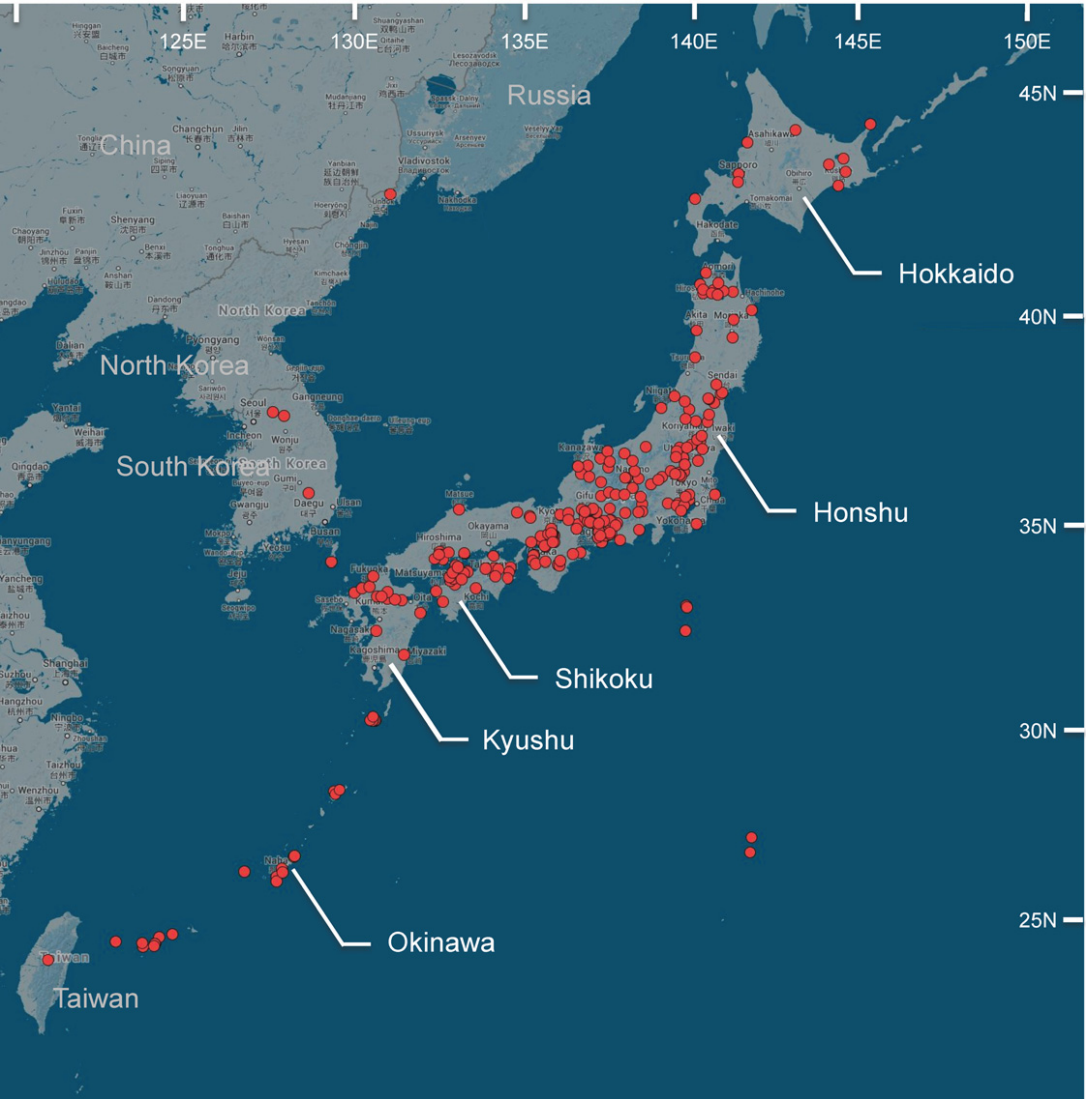
Map of Japan with sampling localities (red circles), created using Google Maps.
