# Supplementary figures and images for: DNA Barcoding of Japanese Click Beetles (Coleoptera, Elateridae)
Source: PLoS One. 2015 Jan 30;10(1):e0116612. doi: 10.1371/journal.pone.0116612 (PMC4312051; doi:10.1371/journal.pone.0116612)

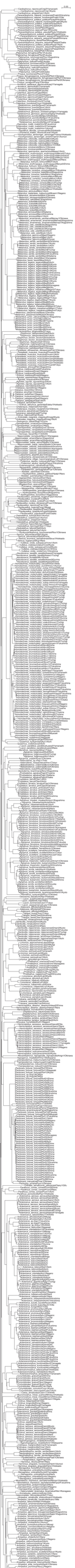

Supplement: S1 Fig — Species (and subspecies) name|Sample ID|Prefecture (or country) are shown. (PDF) [file pone.0116612.s001.pdf]
