# Supplementary material for: DNA Barcoding of Japanese Click Beetles (Coleoptera, Elateridae)
Source: PLoS One. 2015 Jan 30;10(1):e0116612. doi: 10.1371/journal.pone.0116612 (PMC4312051; doi:10.1371/journal.pone.0116612)
Supplement: S2 Fig — (PDF) [file pone.0116612.s002.pdf]

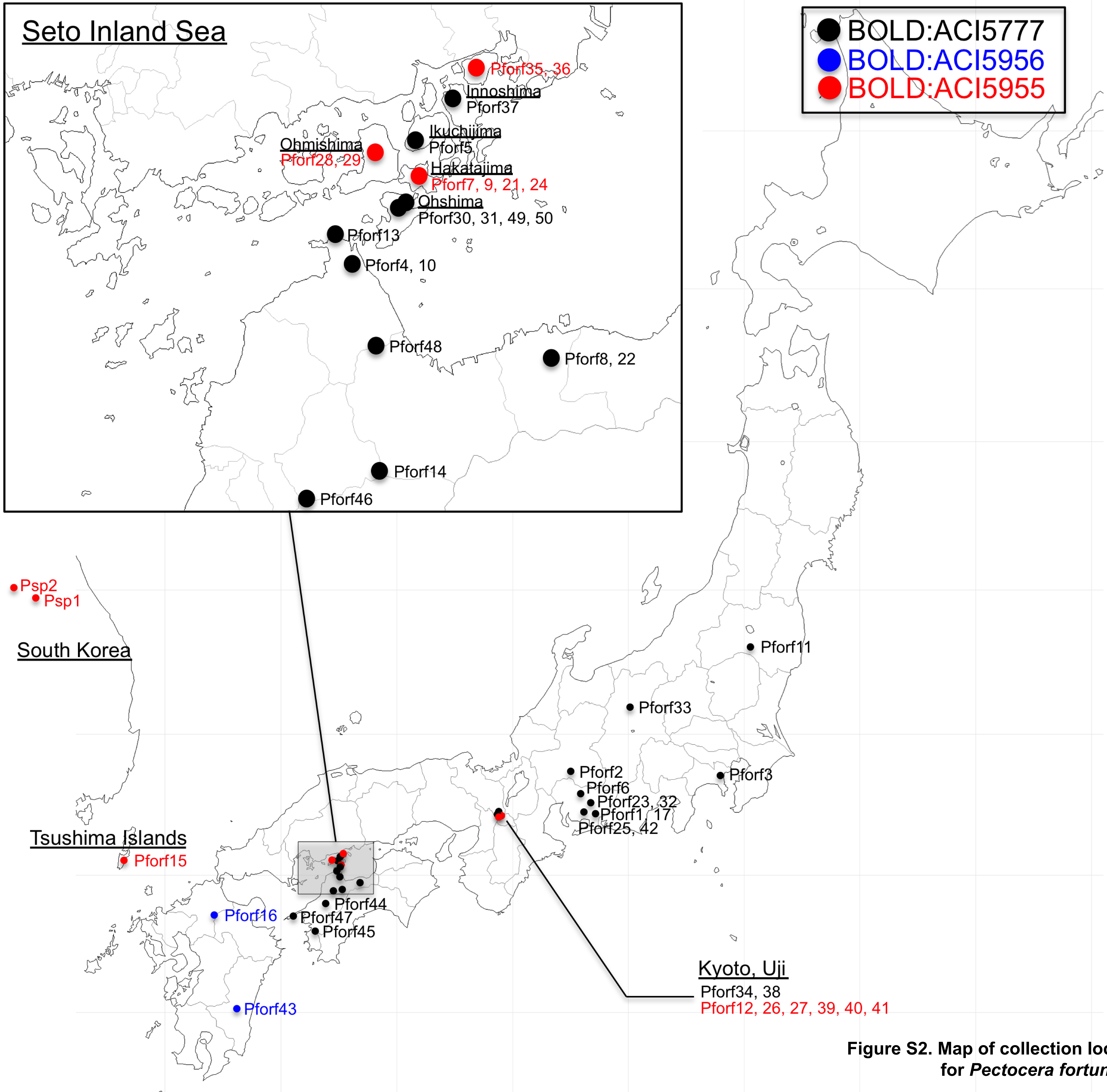

Figure S2. Map of collection localities with sample IDs for *Pectocera fortunei* analyzed in this study.
