# Supplementary material for: DNA Barcoding of Japanese Click Beetles (Coleoptera, Elateridae)
Source: PLoS One. 2015 Jan 30;10(1):e0116612. doi: 10.1371/journal.pone.0116612 (PMC4312051; doi:10.1371/journal.pone.0116612)

Figure S3. Map of collection localities with sample IDs and BINs for *Actenicerus* '*orientalis* group' analyzed in this study.

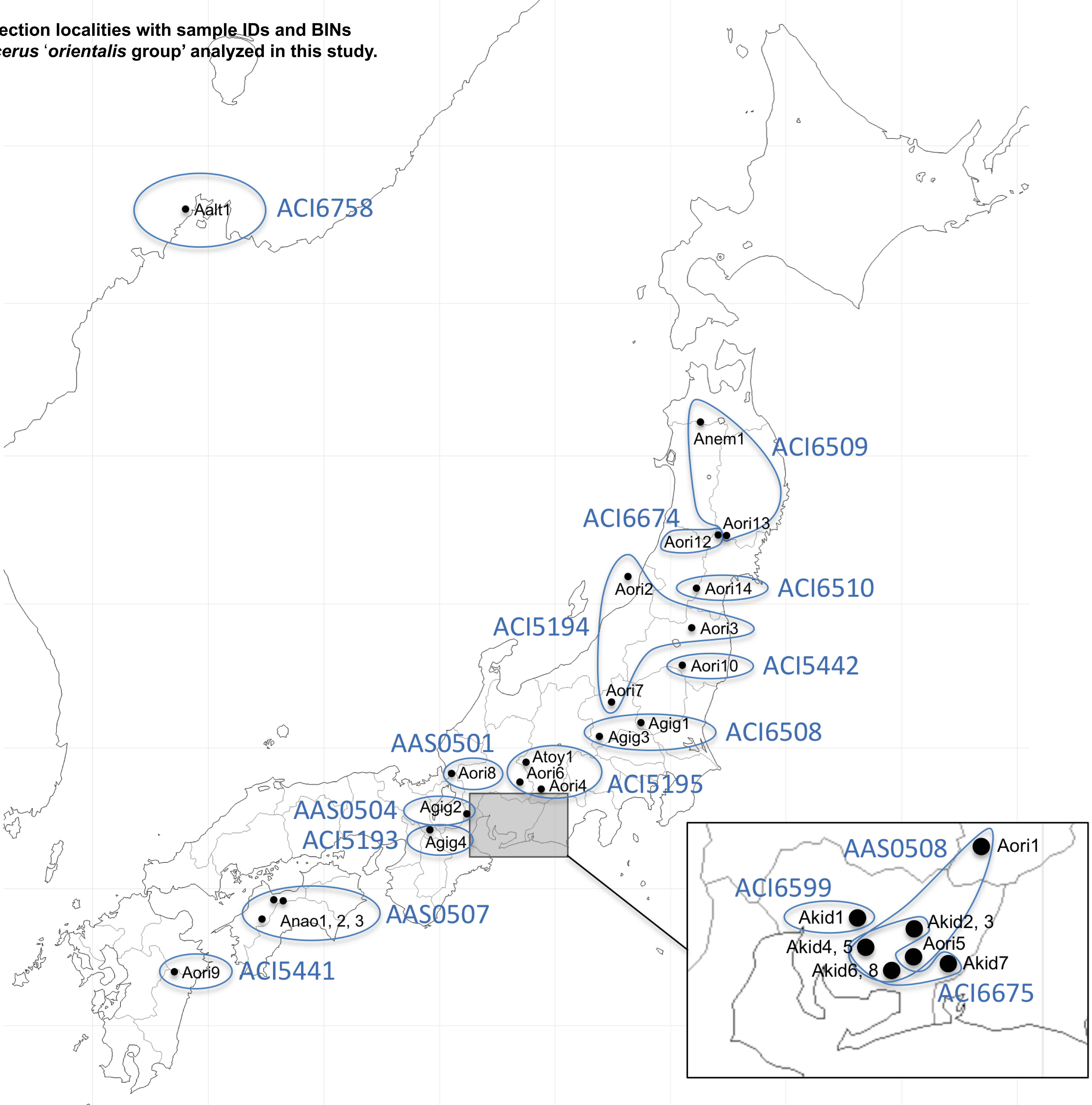

Supplement: S3 Fig — (PDF) [file pone.0116612.s003.pdf]
