# Supplementary material for: DNA Barcoding of Japanese Click Beetles (Coleoptera, Elateridae)
Source: PLoS One. 2015 Jan 30;10(1):e0116612. doi: 10.1371/journal.pone.0116612 (PMC4312051; doi:10.1371/journal.pone.0116612)

Figure S4. Distributions of maximum intraspecific *p*-distance (red) and the nearest neighbor distance for each species (green).

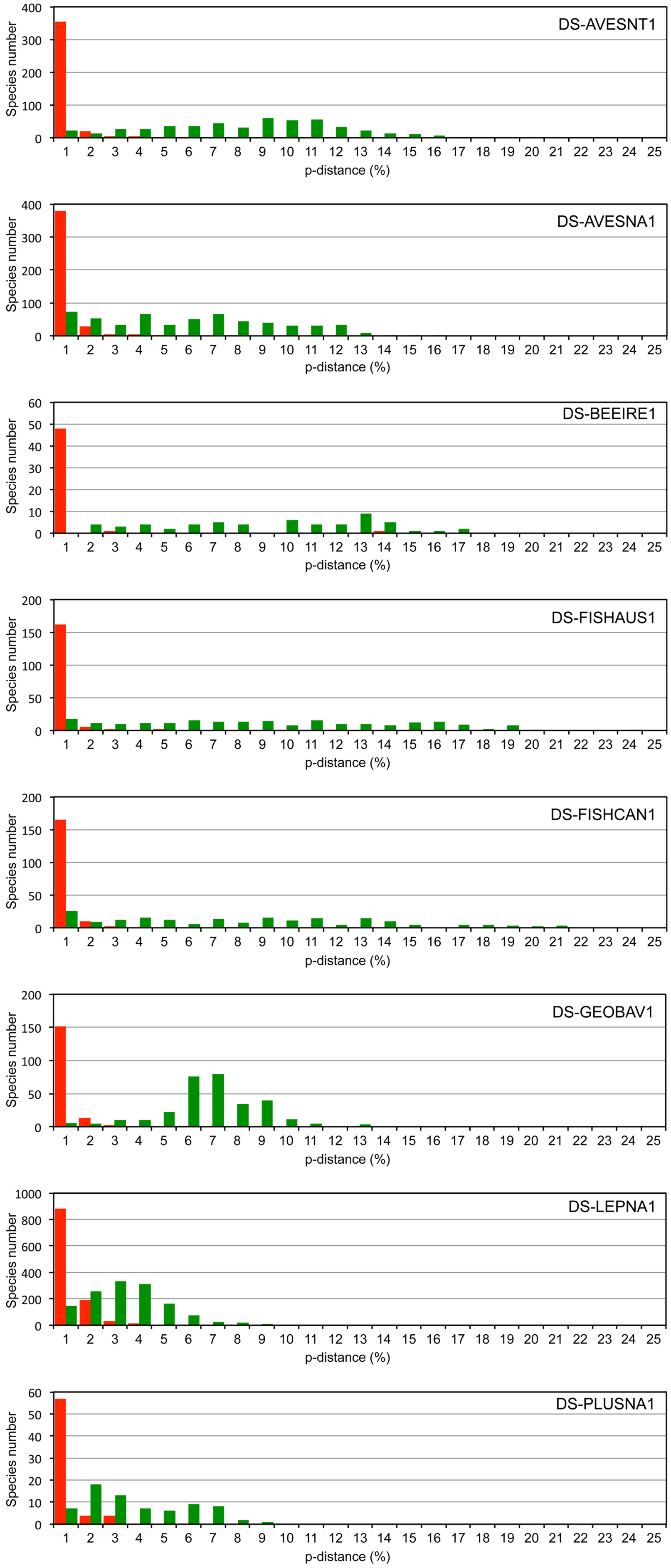

Supplement: S4 Fig — Sequences below 500 bp were eliminated. Eight public DNA barcoding datasets were chosen following Ratnasingham and Hebert, 2013 [32]. DS-AVESNT1, dataset of Neotropical birds (497 species); DS-AVESNA1, dataset of North American birds (575 species); DS-BEEIRE1, dataset of Irish bees (58 species); DS-FISHAUS1, dataset of Australian fishes (214 species); DS-FISHCAN1, dataset of Canadian fishes (190 species); DS-GEOBAV1, dataset of German geometer moths (301 species); DS-LEPNA1, dataset of North American moths and butterflies (1347 species); and DS-PLUSNA1, dataset of North American noctuid moths in the Plusiinae subfamily (71 species). Public data were acquired from the BOLD system on 7 April 2014. (PDF) [file pone.0116612.s004.pdf]
